# Bacteriophage-Based Detection of *Staphylococcus aureus* in Human Serum

**DOI:** 10.3390/v14081748

**Published:** 2022-08-10

**Authors:** Matthew Brown, Alex Hall, Henriett Zahn, Marcia Eisenberg, Stephen Erickson

**Affiliations:** 1 Laboratory Corporation of America Holdings, Burlington, NC 27215, USA; 2 Laboratory Corporation of America Holdings, New Brighton, MN 55112, USA

**Keywords:** phage-based detection, *Staphylococcus aureus*, bacteriophage, luciferase reporter phage, phage therapy, serum

## Abstract

Bacteriophages have been investigated for clinical utility, both as diagnostic tools and as therapeutic interventions. In order to be applied successfully, a detailed understanding of the influence of the human matrix on the interaction between bacteriophage and the host bacterium is required. In this study, a cocktail of luciferase bacteriophage reporters was assessed for functionality in a matrix containing human serum and spiked with *Staphylococcus aureus*. The inhibition of signal and loss of sensitivity was evident with minimal amounts of serum. This phenotype was independent of bacterial growth and bacteriophage viability. Serum-mediated loss of signal was common, albeit not universal, among *S. aureus* strains. Immunoglobulin G was identified as an inhibitory component and partial inhibition was observed with both the f(ab’)_2_ and Fc region. A modified bacteriophage cocktail containing recombinant protein A was developed, which substantially improved signal without the need for additional sample purification. This study highlights the importance of assessing bacteriophage activity in relevant host matrices. Furthermore, it identifies an effective solution, recombinant protein A, for promoting bacteriophage-based detection of *S. aureus* in matrices containing human serum.

## 1. Introduction

The commercial application of bacteriophages (phages) for detection, prevention, and treatment of bacterial contamination has shown significant promise over recent years. Phage-based detection methods, for example, have been designed to promote food safety, biodefense, and identification of infectious agents in both humans and animals [1,2,3,4,5]. Preventative application of phages to food, animal feed, and medical products has also been explored [6,7,8]. Therapeutic interventions using phages to treat plant, animal, and human diseases continue to be pursued, and may be beneficial in addressing emerging antibiotic-resistant pathogens [9,10,11]. The broad potential of these technologies has generated a concomitant need to understand phage performance in biologically complex matrices.

Although phages are generally robust in their capacity to infect a bacterial host, evidence of inhibition in clinical matrices has been observed. This was first noted a century ago, not long after the discovery of phage themselves, when animal serum was found to inhibit lytic activity of staphylococcal phages [12]. Shortly thereafter, work confirmed this finding in human serum and it was implicated in the failure of some of the earliest attempts at using phages therapeutically against staphylococcal infections [13]. Importantly, inhibition is not a fundamental property of all phages and is, at least partially, specific for staphylococcal phage. This has been demonstrated with side-by-side testing of matrices containing plasma where inhibition of *Staphylococcus aureus* phages but not *Pseudomonas aeruginosa* or *Enterococcus faecalis* phages was observed [14,15]. Nonetheless, this inhibitory activity continues to complicate modern attempts at developing phage-based applications targeting *S. aureus*, a major clinical pathogen. For example, human blood was recently found to prevent the killing of *S. aureus* by two commercially available phages, SATA-8505 and Phage K [16,17]. Furthermore, efforts to address specific *S. aureus* diseases, including mastitis and infective endocarditis, with phages have also encountered similar issues [15,18]. Loss of activity in some clinical matrices is, thus, a potential barrier to the effective use of phage-based technologies, including diagnostics and therapeutics, to address public health concerns.

Efforts to identify the source of inhibition have generally focused on staphylococcal phages in clinical matrices containing blood and blood derivatives. In this regard, inhibition of staphylococcal phage activity has been observed across blood, plasma, and serum preparations, and persists after incubation at 56 °C [17,19]. Cellular components (only present in whole blood), clotting factors (absent in serum), and complement (inactivated by heating) are thus not essential to this phenotype. Several studies have directly implicated immunoglobulins as the primary inhibitory component. Evidence to support this includes that the activity is concentrated in the gamma globulin serum fraction, can be adsorbed by *S. aureus* expressing protein A, and can be partially recapitulated by immunoglobulin G (IgG) alone [19,20,21]. In summary, this inhibition of phage activity, thus far specific to staphylococcal phage, is seemingly driven by the presence of immunoglobulins in clinical matrices.

The absence of effective phage performance in clinical matrices is not associated with the direct neutralization of either the staphylococcal phage or the bacterium [17,19,21]. Given this, aspects of the host and phage interaction, such as phage attachment, expression of the phage genome, or host cell lysis are likely being affected by the presence of immunoglobulins. Inhibition was initially suggested to occur downstream of phage attachment, as the loss of phage replication and lysis did not correspond with a significant decrease in adsorption [19]. A later study, however, reported a significant impact on adsorption by serum and hypothesized that immunoglobulins may compete with phage for bacterial surface receptors [21]. Differences in experimental design, bacterial strains, and phages may account for these differing conclusions and additional studies will be needed to further clarify the underlying mechanism.

Phage-based diagnostics are also likely to encounter similar clinical matrices. Luciferase phage reporters are a commonly used approach among phage-based diagnostics, employing a recombinant phage encoding a luciferase gene [22]. Here, the luciferase gene is expressed only following infection of viable host and can be used to sensitively and accurately identify the presence of a target bacterial species. The expression of phage-encoded reporters during infection in clinical matrices is, thus, essential to real-world assay performance.

The purpose of the present study was to assess the performance of a previously published staphylococcal luciferase phage reporter cocktail in human serum [23]. Inhibition of the phage-encoded luciferase signal was evident, and detection of various *S. aureus* strains was significantly impeded by the presence of serum. IgG was identified as the causative agent of this phenotype, capable of facilitating significant inhibition alone. The development of a novel phage cocktail, including recombinant protein A to neutralize IgG, yielded promising results, overcoming significant inhibition, and demonstrating a restored ability to sensitively detect *S. aureus* in serum. The results of this study have important implications broadly in phage-based applications and highlight the importance of the clinical matrix on the interaction between phage and bacterium.

## 2. Materials and Methods

### 2.1. Bacterial Strains and Bacteriophage Reporter Cocktail

All strains of *S. aureus* used in this study were commercially obtained from the American Type Culture Collection (ATCC) (Manassas, VA, USA). Strain designations and nomenclature are based on information provided by ATCC. Bacterial strains were routinely cultured at 37 °C in tryptic soy broth (TSB) (Oxoid, Hampshire, UK) with shaking at 250 revolutions per minute (RPM).

The generation, preparation, and performance of the two-phage reporter cocktail utilized in this study has been previously described in detail [23]. Briefly, two broad-host-range staphylococcal phages were engineered by homologous recombination to encode NanoLuc^®^, a genetically modified luciferase with robust signal generation and attractive kinetics [24]. The *S. aureus* strain ATCC 12600 was used to generate high titer lysates, which were subsequently purified on a cesium-chloride gradient. For this study, a working stock of the phage cocktail was prepared from this material in SM buffer (50 mM Tris–HCl pH 7.5, 8 mM MgSO_4_·7H_2_O, 100 mM NaCl, and 0.01% (*w*/*v*) gelatin) and contained each recombinant phage at 1.6 × 10^8^ plaque forming units (PFU) per mL.

### 2.2. Phage-Based Detection of S. aureus

Phage-based detection of *S. aureus* was routinely performed as previously described with slight modification [23]. Overnight bacterial cultures were diluted in TSB to the desired number of colony forming units (CFU) based on an optical density (600 nm) of one being equivalent to roughly 8 × 10^8^ CFU/mL. When indicated, bacterial burdens were confirmed by plating in at least duplicate on TSB agar (TSA). Diluted bacterial cultures were added to triplicate wells on a 96-well strip plate (Greiner Bio-One GmbH, Frickenhausen, Germany). Wells containing sterile TSB were included to assess assay background without bacteria. Well volume at the start of the assay was 150 µL. Sample-containing strip plates were sealed with cover film (Thermo Fisher Scientific, Rochester, NY, USA) and incubated for 4 h at 37 °C. Although enrichments are not strictly necessary for phage-based detection, this procedure was chosen to mimic the conditions of the original study. Following this enrichment, 10 µL of phage cocktail working stock was added to each well to obtain a final concentration of approximately 1 × 10^7^ PFU/mL. Strip plates were sealed once again and incubated for an additional 2 h at 37 °C to facilitate infection and production of phage-encoded luciferase. After incubation, the presence of luciferase was determined by the addition of 65 µL of detection solution, a master mix roughly consisting per well of 50 µL Nano-Glo^®^ buffer (Promega, Madison, WI, USA), 15 µL TSB, and 1 µL Nano-Glo^®^ substrate (Promega, Madison, WI, USA). Light production was then quantified as relative light units (RLU) using a GloMax^®^ Navigator (Promega, Madison, WI, USA) with a 3 min delay and a 1 s integration time.

### 2.3. Phage-Based Detection of S. aureus in the Presence of Serum and Serum Proteins

Assay performance in the presence of serum and serum proteins was determined using a modification of the method described in Section 2.2. Cultures of *S. aureus* (ATCC 12600) were first diluted in TSB to obtain the desired number of CFU in 75 µL and then added to triplicate wells per planned condition. An equivalent volume (75 µL) of either TSB or each serum/serum protein diluted in TSB, was subsequently added to achieve the specific concentration in a 150 µL well volume. Off-the-clot normal human serum, non-denatured albumin from human serum, and whole IgG from human serum were obtained from Millipore Sigma (Burlington, MA, USA). Plasma-derived human AB serum was obtained from Corning (Corning, NY, USA). Human IgG fragments (Fc and F(ab’)_2_) were obtained from Rockland (Limerick, PA, USA). When indicated, sodium azide (Millipore Sigma, Burlington, MA, USA) was also included to standardize the levels of this preservative, present in the commercially obtained IgG fragments, across samples. Enrichment, phage infection, and detection were unmodified and performed, as described in Section 2.2.

### 2.4. Assessment of Bacterial Growth and Phage Cocktail Viability in the Presence of Serum

To evaluate the effect of human serum on bacterial enrichment, a modification of the method described in Section 2.2. was performed. *S. aureus* (ATCC 12600) cultures were diluted and mixed with either TSB or diluted off-the-clot human serum in triplicate wells, as previously described. The bacterial dilution was plated in triplicate on TSA to establish the starting CFU (Time 0 h). The plate was sealed and enriched at 37 °C for 4 h, mimicking routine enrichment conditions. After this enrichment, each of the triplicate wells were mixed, diluted, and plated on TSA in duplicate to quantify bacterial growth in each condition. CFU were counted following overnight incubation at 37 °C.

To assess the impact of human serum on the viability of the phage cocktail, PFU were compared following a mock infection in either TSB or diluted off-the-clot human serum. TSB was mixed with serum to the indicated concentrations in triplicate wells (150 µL well volume). Phage working stock (10 µL) was immediately added to each well. Wells were sealed and incubated at 37 °C for 2 h, to mimic routine infection. After this 2 h period, wells were mixed, diluted, and assessed for PFU using a double agar overlay of 0.3% TSA semi-solid atop TSA. Log phase *S. aureus* (ATCC 12600) was used as the host strain for plaque formation and plaques were counted following overnight incubation at 37 °C.

### 2.5. Neutralization of Serum by Native and Recombinant Protein A

To assess the ability of protein A to neutralize the inhibitory effects of serum on phage-based detection, a modification of the method described in Section 2.2. was performed. Native protein A from *S. aureus* (Millipore Sigma, Burlington, MA, USA) or recombinant protein A Cys (C-Term) produced in *E. coli* (Novus Biologicals, Littleton, CO, USA) were obtained and stocks prepared in sterile water, as per manufacturer’s instructions. Bacterial cultures of *S. aureus* (ATCC 12600) were diluted, added to wells at a volume of 75 µL, and plated to confirm CFU, as previously described. In these experiments, 65 µL of diluted off-the-clot human serum or TSB was added to each well. Native protein A, recombinant protein A, or sterile water was then added in a volume of 10 µL to achieve a final well volume of 150 µL. Triplicate wells were prepared for each condition. Enrichment, phage infection, and detection were unmodified and performed, as described in Section 2.2.

### 2.6. Detection of S. aureus in Serum Using a Modified Phage Cocktail

The method described in Section 2.2. was once again utilized with modifications to determine the utility of a phage cocktail containing recombinant protein A. Dilutions of *S. aureus* (ATCC 12600) were prepared, mixed with TSB or diluted off-the-clot human serum, and enriched for 4 h, as previously described. For these experiments, the standard phage cocktail was modified to contain either recombinant protein A (4.55 mg/mL) or an equivalent volume of sterile water. After the 4 h enrichment, phage infection was initiated by adding 11 µL of either phage cocktail, achieving similar phage concentration and a recombinant protein A concentration of roughly 0.3 mg/mL in each well when included. Plates were sealed and incubated for a 2 h infection period at 37 °C before detection of luminescence was performed, as previously described in Section 2.2.

### 2.7. Statistical Analysis, Figure Preparation, and Reference Management

The statistical analysis of results was performed using GraphPad Prism^®^ 9 (GraphPad Software, San Diego, CA, USA). Unless otherwise indicated, significance was determined by a two-way analysis of variance (ANOVA) with a Bonferroni’s multiple comparisons test. Figure preparation was conducted using GraphPad Prism^®^ 9 and Microsoft Word 2019 (Microsoft Corporation, Redmond, WA, USA). References were managed using EndNote^®^ 20 (Clarivate, London, UK).

## 3. Results

### 3.1. Human Serum Potently Inhibits Phage-Based Detection of Staphylococcus aureus

To determine the impact of human serum on phage-based diagnostics for *S. aureus*, a previously developed screen was re-examined [23]. Bacterial detection by this method is mediated by an engineered cocktail of luciferase (NanoLuc^®^) encoding phages, which generates a robust signal following infection of its bacterial host. Although not essential for detection, enrichment prior to phage infection was previously used to improve assay sensitivity and, in the presence of antibiotics, identify antimicrobial resistance. In the present study, detection of *S. aureus* was investigated using a similar approach consisting of a four-hour sample enrichment, a two-hour phage infection, and a brief detection step via a luminometer.

**Figure 1:**
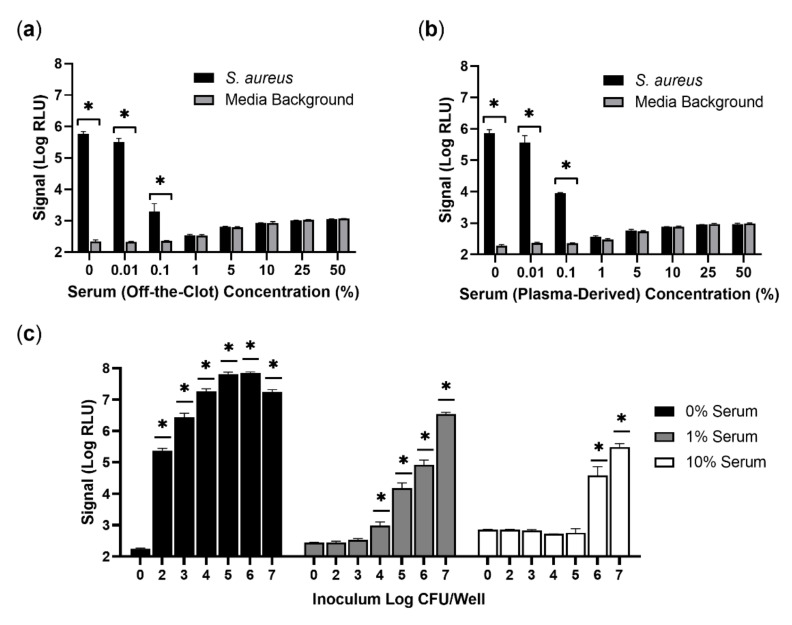
Inhibition of bacteriophage-based detection of *S. aureus* by human serum. Off-the-clot serum (**a**) and plasma-derived serum (**b**) were diluted in TSB (media background) or TSB spiked with approximately 100 CFU of *S. aureus* (ATCC 12600). In (**c**), three concentrations of off-the-clot serum were assessed similarly with increasing bacterial burdens. In each case, luciferase production was evaluated following a 4 h enrichment and a 2 h infection and is provided as log-transformed relative light units (Log RLU). Error bars represent standard deviation from triplicate wells. A two-way ANOVA with a Bonferroni’s multiple comparisons test was performed, comparing each spiked condition to the corresponding media background without *S. aureus*. Asterisks (*) indicate significance (*p* < 0.05).

In agreement with prior performance, a substantial signal, provided as log-transformed relative light units (Log RLU), was detected from approximately 100 colony forming units (CFU) of *S. aureus* (ATCC 12600) in samples without human serum (Figure 1). This was easily distinguishable from the media background without bacteria. In the presence of increasing amounts of human serum, however, signal production was thoroughly quenched. A dose-dependent phenotype was observed with serum from both commonly used sources, off-the-clot (Figure 1a) and plasma-derived (Figure 1b). The presence of only 1% human serum from either source was sufficient to reduce the signal obtained by three logs, making it indistinguishable from background. The media background itself was also affected, albeit to a lesser extent, demonstrating up to a five-fold increase at 50% human serum. Untransformed RLU and CFU data are provided (Table S1).

Given the similarity in performance, off-the-clot serum was used for all further experiments. To determine if serum inhibition was dependent on the number of bacterial cells being detected, burdens up to 10 million CFU were tested (Figure 1c). Without serum, the signal initially increased proportionally with CFU, leveling off at about 1 million CFU and demonstrating a slight reduction at roughly 10 million CFU. This hook effect-like decrease may be due to suboptimal protein production in stationary phase cells following a 4 h enrichment with very high burdens. In contrast to this sensitivity, a weak signal over the background could only be detected in 1% serum when approaching 10,000 CFU, while approximately 1 million CFU were required to generate a signal over background in 10% human serum. Untransformed RLU and CFU data are provided (Table S2). These results suggest that matrices containing human serum will be problematic to phage-based detection of *S. aureus*. The presence of even low amounts of serum (1%) resulted in significant loss in assay sensitivity and reduced limit of detection.

### 3.2. Serum Inhibition of Phage-Based Detection Is Common among S. aureus Strains

**Figure 2:**
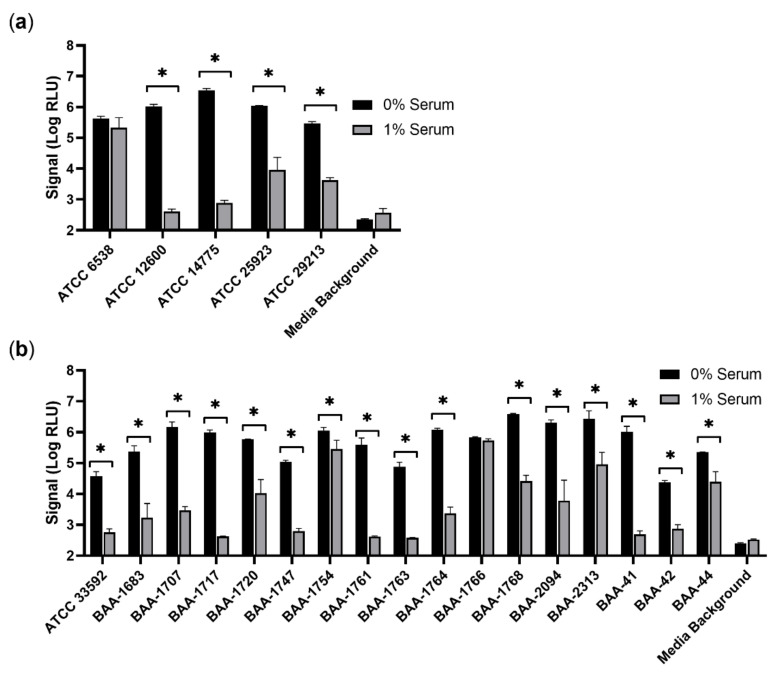
Phage-based reporter signal is reduced by serum in many *S. aureus* strains. A diverse panel of MSSA (**a**) and MRSA (**b**) strains was evaluated for luciferase production following a 4 h enrichment and a 2 h infection in the presence or absence of 1% off-the-clot human serum. Initial inoculum was approximately 100 CFU per well. Signal is provided as log-transformed relative light units (Log RLU). Error bars represent standard deviation from triplicate wells. A two-way ANOVA with a Bonferroni’s multiple comparisons test was performed, comparing 0% and 1% serum conditions for each sample. Asterisks (*) indicate significance (*p* < 0.05).

Although the detrimental effect of human serum on phage-based detection of ATCC 12600, a methicillin-sensitive *S. aureus* (MSSA), was clear, it was unknown if this phenotype would be common across diverse *S. aureus* strains. A panel of 22 *S. aureus* strains from the American Type Culture Collection (ATCC), including 4 additional MSSA strains and 17 methicillin-resistant *S. aureus* (MRSA) was thus examined at a burden of approximately 100 CFU. Of note, the MRSA strains represent significant diversity and include 7 different SCCmec types and 10 different USA types, according to their source. In agreement with prior work, all 22 *S. aureus* strains yielded strong signal in conditions without human serum (Figure 2) [23]. The lowest signal obtained from any strain in media alone was greater than 20,000 RLU, nearly two logs above the corresponding background. As seen previously, the inclusion of only 1% human serum dramatically reduced signal from ATCC 12600. A reduction in signal was also observed in three of the other four MSSA strains tested (Figure 2a). Interestingly, signal from ATCC 6538 appeared to be minimally affected by the inclusion of 1% serum and still generated greater than 100,000 RLU. Similar to MSSA, the majority of MRSA strains (16 of 17) demonstrated a reduction in signal from 1% serum, although the extent of this inhibition varied greatly. As with ATCC 6538, signal following infection of BAA-1766 was seemingly unaffected by 1% serum. Untransformed RLU and CFU data are provided (Table S3). These results suggest that serum-mediated inhibition of phage-based detection is a common, albeit not universal, phenotype among *S. aureus* strains.

### 3.3. Bacterial Growth and Phage Viability Are Unaffected by Human Serum

Serum could feasibly interfere with assay performance by reducing bacterial viability and growth. To determine if sample enrichment was negatively impacted by serum, CFU of ATCC 12600 was evaluated following a 4 h enrichment period with and without human serum. Signal following infection of this *S. aureus* strain had previously been shown to be sensitive to serum. Despite this, similar levels of bacterial growth were observed with either 0, 1, or 10% human serum (Figure S1a). This result indicates that sufficient bacteria for detection are present following enrichment and that reduced signal cannot be explained by this mechanism. Another possible source of assay inhibition is neutralizing anti-phage antibodies. Given that signal production is only minimally affected in some strains, it is unlikely that the phage cocktail, a universally critical reagent, is being targeted. To support this, samples of phage cocktail were incubated with 0, 1, or 10% human serum for 2 h to mimic infection before being plated to determine plaque forming units (PFU). No change in PFU was observed following incubation in serum, suggesting a lack of neutralization (Figure S1b). These findings indicate that bacterial growth and phage viability are not detrimentally impacted by the presence of human serum at these concentrations and cannot explain the interference with phage-based detection.

### 3.4. The Effect of Albumin and Immunoglobulin G on Phage-Based Detection of S. aureus

**Figure 3:**
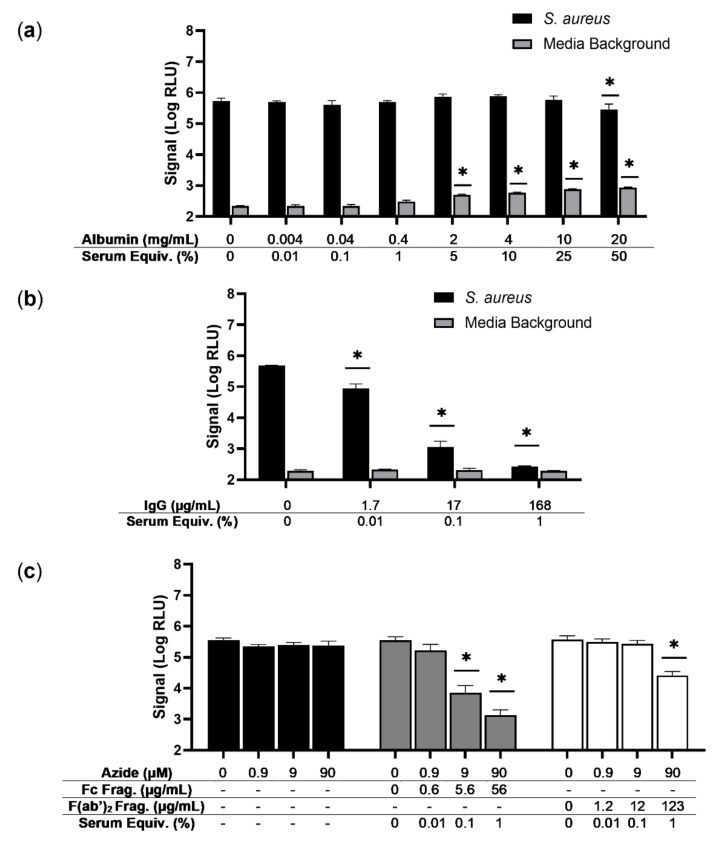
Analysis of serum proteins for inhibition of phage-based detection. Human serum albumin (**a**), human immunoglobulin G (IgG) (**b**), or fragments of human IgG (**c**) were diluted in TSB spiked with approximately 100 CFU of *S. aureus* (ATCC 12600). In (**a**,**b**), media background is the indicated reagent diluted in TSB alone. Luciferase production was evaluated following a 4 h enrichment and a 2 h infection and is provided as log-transformed relative light units (Log RLU). Error bars represent standard deviation from triplicate wells. A two-way ANOVA with a Bonferroni’s multiple comparisons test was performed, comparing the concentration of each serum protein to the corresponding media or buffer control. Asterisks (*) indicate significance (*p* < 0.05). To allow comparison, the approximate percentage of serum that each protein concentration is equivalent to is provided (Serum Equiv.).

In order to determine if the causative component of serum could be identified, two of the most abundant serum proteins, human serum albumin (HSA) and IgG, were evaluated for inhibitory potential. Albumin did not substantially affect signal from *S. aureus*, demonstrating only a minor reduction in signal at high concentrations (20 mg/mL), roughly equivalent to 50% human serum (Figure 3a). Interestingly, background signal increased in a dose-dependent fashion with albumin, mirroring human serum in this regard. Thus, although albumin does not appear to be a major inhibitory component of serum, it is likely responsible for the increased background observed with this matrix. IgG, on the other hand, demonstrated a significant inhibition of signal obtained from *S. aureus* without affecting background signal (Figure 3b). Untransformed RLU and CFU data are provided (Table S4). This dose-dependent phenotype was similar to that of whole serum, providing nearly complete signal quenching at concentrations close to those expected in 1% serum.

To further dissect the inhibition observed with whole human IgG, commercially available preparations of the IgG fc and f(ab’)_2_ region were obtained. These products contained low levels of sodium azide, a preservative, which could feasibly have an independent negative impact on assay performance. As such, concentrations of each IgG fragment were compared to an appropriate buffer control with matching azide concentrations (Figure 3c). The highest concentration of sodium azide (90 µM) had only a minimal effect on signal production from *S. aureus*, generating more than 100,000 RLU. Interestingly, both fragments were found to elicit some degree of signal inhibition. The fc region reduced signal by over two logs at 56 µg/mL while the f(ab’)_2_ region resulted in only a one log reduction in signal at 123 µg/mL. These concentrations are both approximately equivalent to 1% human serum, suggesting a predominate role for the fc region in this phenotype. Similar to whole IgG, neither fragment affected the media background (Figure S2). Untransformed RLU and CFU data are provided (Table S5). Overall, albumin appears responsible for increased background, while IgG, with contribution from both fragments, inhibits *S. aureus* detection.

### 3.5. Neutralizing Serum Inhibition of Phage-Based Detection with Protein A

**Figure 4:**
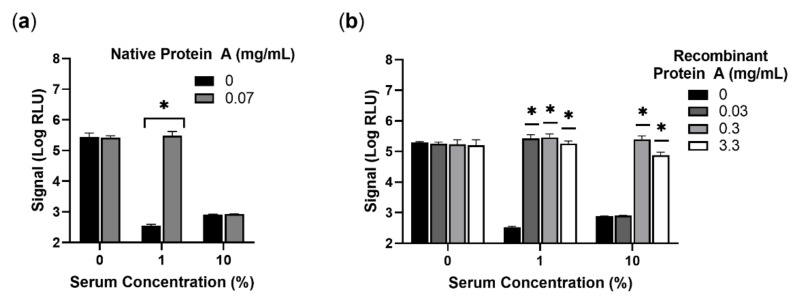
Use of protein A supplementation to recover phage-based signal in human serum. Native protein A from *S. aureus* (**a**), and recombinant protein A produced in *E. coli* (**b**) were diluted in TSB spiked with approximately 100 CFU of *S. aureus* (ATCC 12600). Assay wells also contained either 0, 1, or 10% off-the-clot human serum. Luciferase production was evaluated following a 4 h enrichment and a 2 h infection and is provided as log-transformed relative light units (Log RLU). Error bars represent standard deviation from triplicate wells. A two-way ANOVA with a Bonferroni’s multiple comparisons test was performed, comparing each condition with protein A back to the baseline signal without protein A (0 mg/mL). Asterisks (*) indicate significance (*p* < 0.05).

Given its role in assay interference, neutralization of IgG should alleviate signal inhibition, promoting phage-mediated detection of *S. aureus* in the presence of serum. Protein A is a well-studied surface protein and virulence factor of *S. aureus* that primarily interacts with the Fc region of human IgG [25,26]. Beyond its role in pathogenesis, protein A is widely used industrially to isolate and purify antibodies, with a variety of preparations commercially available. To investigate if protein A could be added to neutralize the inhibitory effect of serum, two products, native protein A from *S. aureus* and recombinant protein A produced in *E. coli*, were obtained. As observed previously, a robust signal was obtained with approximately 100 CFU of *S. aureus* and extensive quenching observed in the presence of human serum (Figure 4). When native protein A from *S. aureus* was included in the assay, the signal was largely restored for conditions with 1% serum (Figure 4a). No signal recovery was observed for 10% human serum, although higher concentrations of protein A could not be reasonably achieved for the native product used in this study. Recombinant protein A was available at substantially higher stock concentrations and allowed for further assessment. As seen with native protein A, recombinant protein A was capable of relieving inhibition on *S. aureus* detection in 1% human serum (Figure 4b). Additionally, recombinant protein A, at higher concentrations, was also capable of alleviating significant inhibition observed from 10% human serum. Importantly, protein A of either variety had no effect on signal without serum, suggesting that the substantial increase in signal was directly related to the loss of serum inhibition. Additionally, neither native nor recombinant protein A had any appreciable effect on media background (Figure S3). Untransformed RLU and CFU data are provided (Table S6). Overall, protein A supplementation demonstrated promise in facilitating phage-based detection of *S. aureus* in serum without the need for any sample purification step.

### 3.6. A Modified Protein A-Containing Phage Cocktail Supports Detection of S. aureus in Serum

**Figure 5:**
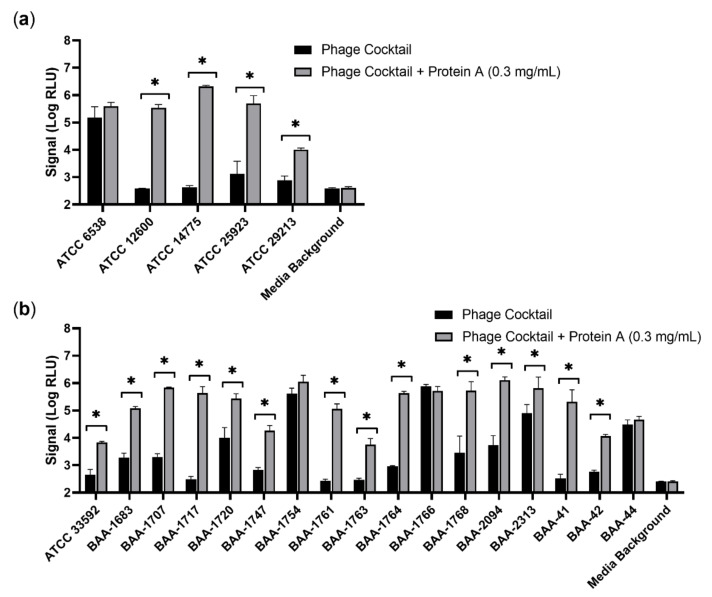
A modified phage cocktail containing recombinant protein A relieves serum suppression of signal broadly among *S. aureus*. A diverse panel of MSSA (**a**) and MRSA (**b**) strains were evaluated for luciferase production following a 4 h enrichment and a 2 h infection in the presence of 1% off-the-clot human serum. Initial inoculum was approximately 100 CFU per well. When indicated, recombinant protein A was included as part of a modified phage cocktail for the 2 h infection only. Signal is provided as log-transformed relative light units (Log RLU). Error bars represent standard deviation from triplicate wells. A two-way ANOVA with a Bonferroni’s multiple comparisons test was performed, comparing each sample in serum with and without protein A. Asterisks (*) indicate significance (*p* < 0.05).

Although protein A was effective when added at the start of the assay, it was of interest to determine if it could be added following bacterial growth in serum. Additionally, detection of only one strain of *S. aureus* (ATCC 12600) in serum had been shown thus far to benefit from protein A. Thus, the panel of 22 *S. aureus* strains was revisited and allowed to enrich in 1% human serum for the typical 4 h period. Following enrichment, a modified phage cocktail containing recombinant protein A was used for the 2 h infection. Based on prior performance, a concentration of 0.3 mg/mL in the well during infection was targeted. In total, 18 of 22 *S. aureus* strains demonstrated a significant increase in signal with the modified cocktail containing protein A (Figure 5). Of note, the four strains unaffected by protein A addition were those that already displayed minimal serum inhibition. This further supports the notion that protein A is specifically reducing the impact of serum on assay performance. Importantly, the lowest signal obtained from any replicate well of any *S. aureus* strain in the presence of 1% human serum with the modified cocktail was 3229 RLU (BAA-1763), roughly 10 times the corresponding media background. Thus, approximately 100 CFU of any *S. aureus* strain in this panel would be detectable in the presence of 1% serum based on previously established cut-offs (two to three times background) [1,23]. Without protein A, however, approximately half of *S. aureus* strains would not be reliably detected above background under these same conditions. Untransformed RLU and CFU data are provided (Table S7). These results highlight the value of recombinant protein A as a supplement to phage cocktails for phage-based detection of *S. aureus* in matrices containing serum.

## 4. Discussion

Although prior studies have observed a negative impact of serum on staphylococcal phage activity, the significance of this phenomenon for phage-based diagnostics, including luciferase phage reporters, had not been previously investigated [17,19,20,21]. In order to evaluate the importance of this interaction, the performance of a Nanoluc^®^-encoding phage cocktail was re-evaluated in the presence of human serum [23]. The serum proved to be a potent inhibitor of *S. aureus* detection by phages (Figure 1a,b). This effect was dose-dependent and significantly diminished the limit of detection of this otherwise sensitive method. Inhibition was not absolute, however, as high burdens of *S. aureus* could still be detected in the presence of serum (Figure 1c). Given these results, the presence of serum in some clinical samples would be expected to, at a minimum, limit the sensitivity of phage-based assays for *S. aureus* detection.

Given the results of this study, it is surprising that one of the few clinically validated phage-based detection methods for *S. aureus* is in a blood-based matrix. Approved by the United States Food and Drug Administration (FDA) in 2011 and produced by Microphage, the KeyPath MSSA/MRSA Blood Culture Test uses bacteriophage infection and amplification, followed by an immunoassay, to detect *S. aureus* in positive BD BACTEC blood cultures [5,27]. To obtain a blood culture with this system, approximately 10 mL of patient blood is mixed with 30–40 mL of bacterial growth media and monitored for carbon dioxide production for up to five days. As a testing matrix, blood cultures, thus, only have 20–25% blood and serum itself represents only roughly half of the blood volume. Importantly, blood cultures using the BACTEC system also do not typically flag as positive until substantial bacterial burdens are present, around 370 million *S. aureus* CFU per mL [28]. Lastly, the KeyPath MSSA/MRSA Blood Culture Test only uses a small (10 µL) sample of the positive blood culture, which is further diluted to an unspecified concentration in a proprietary media during testing [27]. Thus, in agreement with the present study, the impact of serum on phage-based detection of *S. aureus* may be limited when bacterial burdens are high compared to the serum concentration present during infection.

A diverse panel of 22 commercially available isolates was used to determine if the inhibitory effect of serum was universal across *S. aureus* strains. Encompassing both MRSA and MSSA, this panel covered at least 7 SCCmec types and 10 different USA types, according to ATCC. Interestingly, not all *S. aureus* strains were equally affected by serum (Figure 2). Phage-based detection of several strains, such as ATCC 12600, was thoroughly quenched by the presence of just 1% human serum and a loss of 99.9% of signal production was noted. Signal from other strains, such as BAA-1720, experienced a more moderate reduction with some obvious signal persisting over background at these same concentrations. Finally, detection of ATCC 6538 and BAA-1766 was not significantly affected by 1% serum and robust signal was detected regardless of its presence. The mechanism behind this strain-to-strain variation of serum inhibition remains to be elucidated. However, it is clear that detection of *S. aureus* strains by a phage-based method in bacterial media alone may not be sufficient to indicate performance in clinical matrices. Importantly, these results also suggest that diverse panels of *S. aureus* are needed to validate the impact of a clinical matrix on phage-mediated detection.

Due to significant serum sensitivity and high baseline signal, ATCC 12600 was an ideal *S. aureus* strain to further investigate this phenotype. No effect on the bacterial growth of this strain was observed during a 4 h enrichment in serum (Figure S1a). The loss of signal from the phage reporter cocktail is, thus, not due to a bactericidal or bacteriostatic effect of serum on *S. aureus*. This is in agreement with prior work, which found that *S. aureus* viability and growth in serum could not explain a lack of phage propagation and lytic activity [17]. In addition to this, no loss of phage viability was observed following incubation with serum (Figure S1b). This lack of neutralization of phages directly by serum also agrees with prior reports [19]. Given these observations, the negative impact of serum on this reporter cocktail is not due to a direct inhibition of bacteria or phage, but rather related to a disruption of their interaction.

The primary protein components of the serum were investigated to determine if the causative agent of signal inhibition in this assay could be identified (Figure 3). Albumin makes up greater than 50% of protein content in the human serum [29]. Despite this abundancy, albumin had negligible effects on signal production following phage reporter infection (Figure 3a). Similar to serum, a modest increase in background signal was observed with increasing albumin concentrations. Given prior work demonstrating interactions between luciferins and albumin, it is likely that this increased background is luciferase-independent and due to partial albumin-mediated oxidation of the substrate [30,31]. Albumin is not, however, problematic for phage-based detection with this system.

Immunoglobulins are the second most abundant protein in this matrix, with IgG being the predominant class in serum [29]. In the present study, a dose-dependent inhibition of signal from the phage reporter was observed with IgG (Figure 3b). The intensity of this effect mirrored that of serum, approximately a three-log reduction (99.9%) in signal from *S. aureus*. IgG is, thus, capable of mediating the inhibitory effects of human serum on *S. aureus* detection by phage reporters. The whole IgG can be enzymatically separated into functionally distinct fragments, which are commercially available and can be used to provide further insight into this phenotype. The F(ab’)_2_ region of IgG, controlling antigen binding, and Fc region of IgG, mediating host cell receptor and complement protein binding, were probed for inhibitory activity. Surprisingly, both fragments were capable of inhibition of signal following infection with the reporter phage cocktail (Figure 3c). At the equivalent of approximately 1% serum, the F(ab’)_2_ and Fc fragment of human IgG reduced assay signal from *S. aureus* by 89.3% and 99.4%, respectively, compared to a corresponding buffer control. Given the unique properties and functions of these two fragments, it is plausible that the inhibition of phage-based signal by human IgG is multi-faceted, involving distinct mechanisms.

The activity of F(ab’)_2_ against phage-based detection of *S. aureus* may, at least partially, be explained by prior studies on IgG and staphylococcal phage. Serum, whole IgG, and IgG fragments containing the Fab region were found to inhibit staphylococcal phage adsorption [21]. The requirement for antigen-binding regions (Fab) led to the hypothesis that anti-Staphylococcal antibodies may compete with phage for surface receptor accessibility. Anti-staphylococcal IgG antibodies are fairly abundant in healthy human adults and frequently target the cell wall teichoic acid (WTA) of *S. aureus* [32,33,34]. The vast majority of Staphylococcal phage utilize either the WTA backbone or a specific WTA glycosylation moiety as a receptor [35]. Given this overlap, it is plausible that the reduced signal from the reporter phage in the presence of F(ab’)_2_ is due to specific competition for binding of WTA receptors. Future studies may be beneficial in dissecting the definitive contribution of anti-Staphylococcal antibodies to this phenotype.

The Fc fragment of human IgG proved to also be a potent inhibitor of the phage reporter assay, indicating a potential mechanism beyond antigen-binding (Figure 3c). Protein A is an important *S. aureus* virulence factor that is capable of binding to the Fc regions of human IgG to facilitate immune evasion [36,37]. Interestingly, the presence of the cell-wall anchored protein A, itself on the surface of *S. aureus,* has been associated with reduced phage adsorption [38]. Further, studies have revealed that the inhibitory capacity of serum on phage multiplication can be removed by prior adsorption of the serum with *S. aureus*, but only if the strains expressed protein A [20]. Given this, it appears that the loss of signal from the phage reporter is associated with the Fc region of IgG binding to protein A and limiting the accessibility of the bacterial surface. In fact, the inhibition from both fragments of IgG could potentially involve protein A as it has been found to be capable of binding to the F(ab’)_2_ region of the VH3 subgroup of IgG [39]. Overall, future work will be required to clarify the mechanism or mechanisms by which serum IgG inhibits phage-based detection of *S. aureus*.

To achieve detection of *S. aureus* with similar phage-based methods, it appears necessary to either have a large burden of bacteria relative to serum concentration, isolate the bacteria away from the matrix, or neutralize the active component, IgG. Given the suspected role of surface-bound protein A in binding IgG, it was hypothesized that soluble protein A may compete with and neutralize its effect. Indeed, both native and recombinant protein A were capable of neutralizing serum and facilitating phage-based detection of *S. aureus* (Figure 4). Importantly, protein A had no significant effect on detection of *S. aureus* in conditions lacking serum, yet allows the sensitive detection of low burdens of *S. aureus* in 10% human serum. Additionally, detection of 23 of 23 *S. aureus* strains is feasible in matrices containing 1% serum without further optimization (Figure 5). As soluble protein A is added as part of a modified phage cocktail, no additional steps are required, avoiding the need for longer enrichments to achieve high burdens or sample purification to remove IgG. Soluble protein A is thus an attractive commercially available reagent for improving phage detection of *S. aureus* in clinical matrices.

The inhibitory effect of serum investigated herein is relevant to other phage-based applications targeting *S. aureus*, including phage therapy. A recent study found that, despite promising in vitro bactericidal activity, treatment with a staphylococcal phage cocktail alone failed to clear *S. aureus* in a rat model of experimental infective endocarditis [15]. This outcome was suspected to be related to the loss of lytic activity that was subsequently observed during experiments on staphylococcal phage in rat plasma. Although phage-based applications are promising, the results of this study and others suggest caution is needed to ensure the successful translation of in vitro performance. To counteract these concerns, in vitro studies using panels of diverse *S. aureus* strains in clinically relevant matrices can be used to preemptively identify such issues.

In summary, serum was found to be a potent inhibitor of phage-based detection of *S. aureus*. Serum limited the detection of most *S. aureus* strains and broadly reduced the performance of this reporter phage cocktail. No detrimental effect of serum directly on bacterial growth or phage viability was observed. IgG was identified as a causative agent in serum, with inhibitory activity observed with both whole IgG and the Fc and F(ab’)_2_ fragments. Soluble protein A was demonstrated to be capable of neutralizing the effects of serum on *S. aureus* detection and presented itself as a previously undescribed remedy to this matrix issue. Ultimately, a modified phage cocktail containing recombinant protein A was developed, showing promise broadly across *S. aureus* strains without the need for additional purification steps or longer enrichments.

## Data Availability

Untransformed data and CFU values for each figure of the main text can be found in the Supplementary Materials. No materials were generated in this study. Access to the previously described reporter phage cocktail used in this study may require a materials transfer agreement covering potential commercial applications.

## Supplementary Materials

The following supporting information can be downloaded at: https://www.mdpi.com/article/10.3390/v14081748/s1, Figure S1: reduction in signal from serum is not associated with reduced bacterial enrichment or loss of phage viability; Figure S2: media background from IgG fragments (Figure 3c); Figure S3: media background from protein A supplementation (Figure 4); Table S1: loss of assay signal in human serum: RLU and CFU values for Figure 1a,b; Table S2: influence of bacterial burden on serum inhibition: RLU and CFU values for Figure 1c; Table S3: signal inhibition by serum is common across *S. aureus* strains: RLU and CFU values for Figure 2; Table S4: role of serum proteins in signal inhibition: RLU and CFU values for Figure 3a,b; Table S5: role of individual IgG fragments in signal inhibition: RLU and CFU values for Figure 3c and Figure S2; Table S6: protein A supplementation alleviates signal inhibition: RLU and CFU values for Figure 4 and Figure S3; Table S7: a modified phage cocktail allows detection of *S. aureus* in the presence of serum: RLU and CFU values for Figure 5.
