# Supplementary material for: Bacteriophage-Based Detection of Staphylococcus aureus in Human Serum
Source: Viruses. 2022 Aug 10;14(8):1748. doi: 10.3390/v14081748 (PMC9416288; doi:10.3390/v14081748)
Supplement: Supplementary file 1 [file viruses-14-01748-s001.zip › viruses-1854369-supplementary.pdf]

Table S1. Loss of Assay Signal in Human Serum: RLU and CFU Values for Figure 1a and Figure 1b

		Signal (RLU)					
**Serum Source**	**Serum Conc. (%)**	***S. aureus* (111 CFU/Well)[1]**			**Media Background**		
		**Well 1**	**Well 2**	**Well 3**	**Well 1**	**Well 2**	**Well 3**
Off-the-clot	0	620200	661700	467400	251	206	206
	0.01	298800	430600	250600	212	222	212
	0.1	1210	3802	1592	227	237	220
	1	327	361	358	346	357	305
	5	637	622	672	605	650	618
	10	870	867	857	883	911	748
	25	966	997	1055	1065	1098	1051
	50	1101	1070	1161	1156	1185	1178
Plasma-derived	0	648500	606700	985900	198	203	176
	0.01	437200	206200	552100	238	245	220
	0.1	8706	8481	9509	238	221	221
	1	371	397	353	326	278	293
	5	553	581	637	531	586	516
	10	765	768	746	731	810	775
	25	905	896	905	972	957	883
	50	890	991	938	1018	983	955

[1] CFU per well for *S. aureus* strain ATCC 12600 was determined by colony counting and is the average of triplicate plates.

Table S2. Influence of Bacterial Burden on Serum Inhibition: RLU and CFU Values for Figure 1c

S. aureus (CFU/well)[1]	Signal (RLU)								
	0% Serum			1% Serum			10% Serum		
	Well 1	Well 2	Well 3	Well 1	Well 2	Well 3	Well 1	Well 2	Well 3
0	163	181	181	286	263	275	707	742	705
85	244400	195200	272100	275	310	266	727	728	691
850	3227000	2008000	3275000	378	325	322	651	630	726
8500	21890000	15160000	18840000	1033	702	1195	526	523	528
85000	60310000	76480000	62170000	20660	16670	9813	443	786	538
850000	69320000	77460000	67000000	92490	110800	56520	18230	50730	60620
8500000	19750000	14370000	18950000	3048000	3445000	3942000	286000	248900	404300

[1] CFU per well for *S. aureus* strain ATCC 12600 was measured by colony counting and is the average of triplicate plates. This was only directly performed for the 85 CFU/well burden. Other samples were indirectly extrapolated based upon a serial ten-fold dilution scheme using this burden.

Table S3. Signal Inhibition by Serum is Common Across *S. aureus* Strains: RLU and CFU Values for Figure 2

Sample Type	ATCC Strain ID	CFU/well[1]	Signal (RLU)					
			0% Serum			1% Serum		
			Well 1	Well 2	Well 3	Well 1	Well 2	Well 3
MSSA	6538	19	397700	371500	515000	172500	120600	499200
	12600	88	1068000	865800	1203000	391	495	361
	14775	107	4084000	3132000	3205000	672	711	965
	25923	18	1100000	1123000	1058000	3642	9474	23030
	29213	69	252400	320800	316800	3798	5267	3938
MRSA	33592	99	24900	47090	43940	471	758	557
	BAA-1683	60	254000	145900	350600	4013	515	2385
	BAA-1707	79	1613000	1011000	2064000	3926	2291	3020
	BAA-1717	83	764800	1059000	1111000	425	413	431
	BAA-1720	111	583600	600400	583400	28270	3846	11660
	BAA-1747	74	117300	117400	92810	502	777	620
	BAA-1754	109	1498000	1008000	965800	238400	593700	169000
	BAA-1761	63	551800	502000	225100	412	442	402
	BAA-1763	40	52130	86690	97610	376	391	380
	BAA-1764	93	1347000	1198000	1064000	1378	2698	3452
	BAA-1766	105	718100	649900	651100	475300	602600	552900
	BAA-1768	80	3572000	4003000	3853000	42680	18450	22970
	BAA-2094	93	2493000	1716000	2054000	4651	31510	1521
	BAA-2313	81	4720000	2928000	1474000	56600	51620	254900
	BAA-41	51	1255000	1341000	622600	621	517	372
	BAA-42	72	21240	27900	22990	1037	726	596
	BAA-44	71	230900	231100	228700	40640	36570	10630
Media[2]	N/A	N/A	226	236	217	326	296	533
			246	245	268	343	345	326

[1] CFU per well for each *S. aureus* strain was measured by colony counting and is the average of duplicate plates.
[2] Two sets of triplicate media background wells were run. One with the MSSA strains (top) and one with the MRSA strains (bottom).

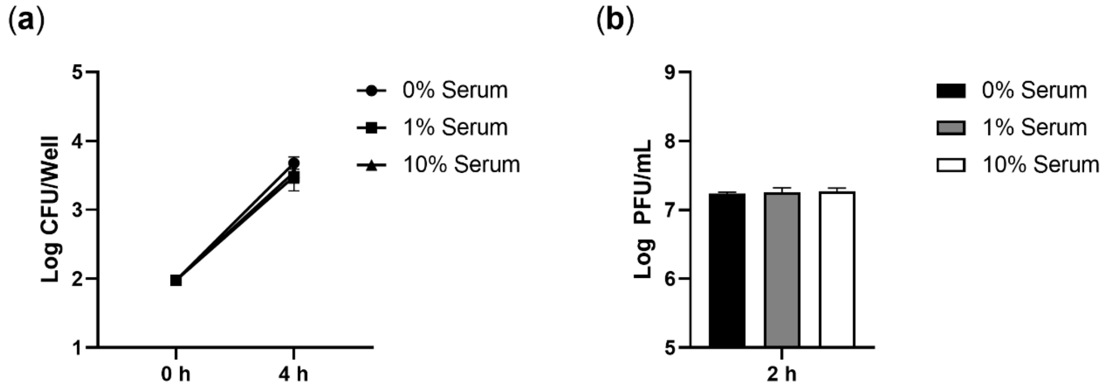

Figure S1. Reduction in signal from serum is not associated with reduced bacterial enrichment or loss of phage viability. (**a**) *S. aureus* strain ATCC 12600 was enriched in the presence of either 0, 1, or 10% human serum. Starting burden (0 h) was approximately 95 CFU per well. Each condition was assayed in triplicate and, following a 4 h enrichment, each well was diluted and spread on duplicate plates to determine CFU. (**b**) Working stock of the phage cocktail was incubated in either 0, 1, or 10% human serum for 2 h in triplicate wells to mimic the infection period. The approximate starting titer in each well was 2 x 10^7 pfu/mL. Following the 2 h mock infection, samples were diluted and the titer for each well was determined by plaque counting. Error bars in each dataset represent standard deviation. A one-way ANOVA with a Bonferroni's multiple comparisons test was performed, comparing conditions with serum to those without serum. No significant ($p < 0.05$) differences were observed for either Log CFU/well or Log PFU/mL.

Table S4. Role of Serum Proteins in Signal Inhibition: RLU and CFU Values for Figure 3a and Figure 3b

Serum Component	Assay Concentration	Signal (RLU)					
		S. aureus (100 CFU/Well)[1]			Media Background		
		Well 1	Well 2	Well 3	Well 1	Well 2	Well 3
Albumin	0 mg/mL	555600	648400	422700	212	226	221
	0.004 mg/mL	541900	508100	456300	242	217	198
	0.04 mg/mL	370300	315200	572200	238	195	232
	0.4 mg/mL	511100	544800	449200	333	312	272
	2 mg/mL	572100	881200	773700	526	503	480
	4 mg/mL	685500	833000	804300	601	617	561
	10 mg/mL	432900	651000	730600	763	743	791
	20 mg/mL	185200	389700	336000	896	847	851
IgG	0 μg/mL	485300	492600	468400	187	213	177
	1.7 μg/mL	128600	79450	71390	211	206	220
	17 μg/mL	1626	723	1306	225	217	178
	168 μg/mL	258	283	257	196	197	190

[1] CFU per well for *S. aureus* strain ATCC 12600 was measured by colony counting and as an average of triplicate plates.

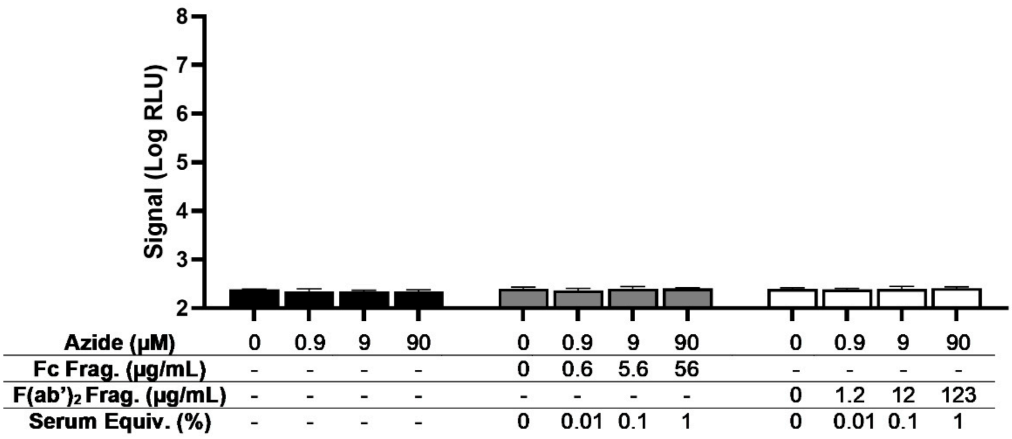

Figure S2. Media background from IgG fragments (Figure 3c). Fragments of human IgG were diluted in TSB and matched to an azide buffer control. Luciferase production was evaluated following a 4 h enrichment and a 2 h infection and is provided as log-transformed relative light units (Log RLU). Error bars represent standard deviation from triplicate wells. A two-way ANOVA with a Bonferroni's multiple comparisons test was performed, comparing the concentration of each serum protein to the corresponding media or buffer control. No significant ($p < 0.05$) differences were observed. For comparison, the approximate percentage of serum that each protein concentration is equivalent to is provided (Serum Equiv.).

Table S5. Role of Individual IgG Fragments in Signal Inhibition: RLU and CFU Values for Figure 3c and Figure S2

Serum Component	Assay Conc. (μg/mL)	Azide Conc. (μM)	Signal (RLU)					
			S. aureus (96 CFU/Well)[1]			Media Background		
			Well 1	Well 2	Well 3	Well 1	Well 2	Well 3
N/A	N/A	0	387000	391600	286700	248	238	235
		0.9	192700	251300	227500	255	205	205
		9	307200	233600	205400	232	213	227
		90	316600	261600	168400	242	216	211
Fc Fragment	0	0	389200	259200	433700	258	233	265
	0.6	0.9	104700	258100	171900	253	210	235
	5.6	9	4002	11700	7623	248	236	280
	56	90	865	1676	1716	258	258	248
$F(ab')_2$ Fragment	0	0	504700	354100	292400	258	250	230
	1.2	0.9	364900	345900	235600	248	251	227
	12	9	361900	240800	223900	285	233	233
	123	90	35600	19930	24290	248	252	277

[1] CFU per well for *S. aureus* strain ATCC 12600 was measured by colony counting and as an average of triplicate plates.

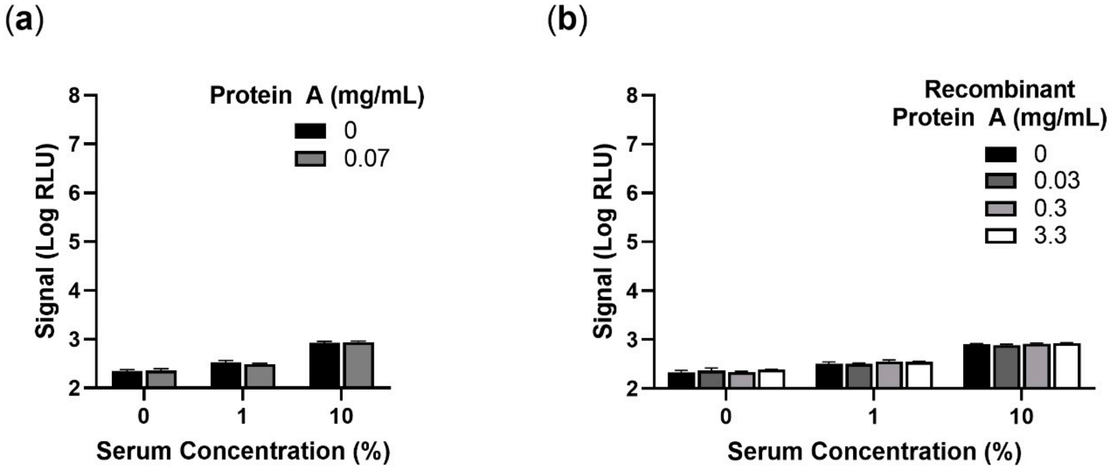

Figure S3. Media background from Protein A supplementation (Figure 4). Native protein A from *S. aureus* **a**), and recombinant protein A produced in *E. coli* (**b**) were diluted in media (TSB). Assay wells also contained either 0, 1, or 10% off-the-clot human serum. Luciferase production was evaluated following a 4 h enrichment and a 2 h infection and is provided as log-transformed relative light units (Log RLU). Error bars represent standard deviation from triplicate wells. A two-way ANOVA with a Bonferroni's multiple comparisons test was performed, comparing each condition with protein A back to the baseline signal without protein A (0 mg/mL). No significant ($p < 0.05$) differences were observed.

Table S6. Protein A Supplementation Alleviates Signal Inhibition: RLU and CFU Values for Figure 4 and Figure S3

Protein A Type	Protein A Conc. (mg/mL)	Serum %	Signal RLU					
			S. aureus[1]			Media Background		
			Well 1	Well 2	Well 3	Well 1	Well 2	Well 3
Native	0	0	193600	315900	341100	235	208	228
		1	387	307	352	342	367	300
		10	846	808	822	857	902	790
	0.07	0	306600	236900	262400	251	222	211
		1	311900	225200	414900	317	317	300
		10	837	820	883	907	836	886
Recombinant	0	0	213100	184100	188500	227	227	190
		1	358	326	307	350	297	318
		10	782	763	770	808	798	832
	0.03	0	170000	168100	206100	207	263	231
		1	366500	232400	220500	308	331	327
		10	818	796	797	748	753	811
	0.3	0	164000	125000	244700	213	217	225
		1	340100	218900	330800	387	342	345
		10	322400	196200	250300	812	866	806
	3.3	0	172800	104500	231100	247	243	243
		1	174900	224800	158200	353	345	361
		10	91620	60430	80840	872	841	831

[1] CFU per well for *S. aureus* strain ATCC 12600 was measured by colony counting and as an average of triplicate plates. The native protein A dataset had 83 CFU/well, while the recombinant protein A dataset had 105 CFU/well.

Table S7. A Modified Phage Cocktail Allows Detection of *S. aureus* in the Presence of Serum: RLU and CFU Values for Figure 5

Sample Type	ATCC Strain ID	CFU/well[1]	Signal (RLU) in 1% Serum					
			Phage Cocktail			Phage Cocktail + Protein A[2]		
			Well 1	Well 2	Well 3	Well 1	Well 2	Well 3
MSSA	6538	32	53080	242300	273400	277100	455100	502600
	12600	109	395	388	386	407900	250500	399800
	14775	94	405	370	503	2089000	2293000	1904000
	25923	59	422	3449	1577	779800	667800	230900
	29213	54	1056	825	516	10630	11400	8814
MRSA	33592	154	276	531	641	6333	6493	7594
	BAA-1683	77	2102	2602	1260	140900	103800	120000
	BAA-1707	96	1725	2740	1775	644800	711000	702200
	BAA-1717	108	401	286	261	439100	261300	744800
	BAA-1720	105	5433	6945	27040	189100	420100	259700
	BAA-1747	79	760	543	758	29660	12670	16420
	BAA-1754	109	414800	655800	264000	661400	1966000	1071000
	BAA-1761	101	270	235	311	74570	173500	115700
	BAA-1763	54	277	345	257	3229	7846	7458
	BAA-1764	89	913	961	841	367000	411700	514400
	BAA-1766	78	788200	884500	650500	423900	797200	427000
	BAA-1768	55	3921	9697	621	311600	1247000	396500
	BAA-2094	93	10490	6722	2178	1467000	1544000	924300
	BAA-2313	90	36660	83060	160100	674700	262100	1664000
	BAA-41	98	495	261	275	149000	649700	95200
	BAA-42	44	577	661	485	12840	12380	9816
	BAA-44	122	20240	31810	44600	34450	55170	54420
Media[3]	N/A	N/A	415	381	393	398	450	356
			261	253	265	277	248	248

[1] CFU per well for each *S. aureus* strain was measured by colony counting and is the average of duplicate plates.
[2] Recombinant protein A at 0.3 mg/mL in the well during infection.
[3] Two sets of triplicate media background wells were run. One with the MSSA strains (top) and one with the MRSA strains (bottom).
